# Conceptualization, detection, and management of psychological distress and mental health conditions among people with tuberculosis in Zambia: a qualitative study with stakeholders’ and TB health workers

**DOI:** 10.1186/s13033-022-00542-x

**Published:** 2022-07-12

**Authors:** T. Mainga, M. Gondwe, R. C. Stewart, I. Mactaggart, K. Shanaube, H. Ayles, V. Bond

**Affiliations:** 1 Zambart, School of Public Health, University of Zambia, Ridgeway, Zambia; 2 Department of Global Health and Development, Faculty of Public Health and Policy, London School of Hygiene and Tropical Medicine, London, UK; 3 Department of Clinical Research, Faculty of Infectious and Tropical Diseases, London School of Hygiene and Tropical Medicine, London, UK; 4 Division of Psychiatry, Centre for Clinical Brain Sciences, University of Edinburgh, Edinburgh, UK; 5 Malawi Epidemiology and Intervention Research Unit (MEIRU), Lilongwe, Malawi

**Keywords:** Tuberculosis, Health workers, TB stakeholders, Mental health, Management, Conceptualisation

## Abstract

**Background:**

In recent years, there has been increased recognition of the need to integrate mental health services into routine tuberculosis (TB) care. For successful integration, policymakers need to first understand the practices of TB health workers in the management of mental health conditions, including depression, anxiety, and psychological distress, and use this to decide how best mental health services could be delivered in tandem with TB services. In this qualitative study we aimed to understand how TB health workers and other stakeholders viewed mental health conditions linked to TB and how they screened and treated these in their patients.

**Methods:**

The study draws on qualitative data collected in 2018 as part of the Tuberculosis Reduction through Expanded Antiretroviral Treatment and Screening for active TB trial (TREATS), conducted in eight urban communities in Zambia. Data were collected through 17 focus group discussions with local health committee members (n = 96) and TB stakeholders (n = 57) present in the communities. Further in-depth interviews were held with key TB health workers (n = 9). Thematic analysis was conducted.

**Results:**

TB stakeholders and health workers had an inadequate understanding of mental health and commonly described mental health conditions among TB patients by using stigmatizing terminology and overtones, for example *“madness”,* which often implied a characterological flaw rather an actual illness. Psychological distress was also described as “*overthinking”,* which participants attributed to psychosocial stressors, and was not perceived as a condition that would benefit from mental health intervention. There were no standard screening and treatment options for mental health conditions in TB patients and most TB health workers had no mental health training. TB Stakeholders and health workers understood the negative implications of mental health conditions on TB treatment adherence and overall wellbeing for TB patients.

**Conclusions:**

TB stakeholders and health workers in Zambia have a complex conceptualisation of mental health and illness, that does not support the mental health needs of TB patients. The integration of mental health training in TB services could be beneficial and shift negative attitudes about mental health. Further, TB patients should be screened for mental health conditions and offered treatment.

*Trial registration number* NCT03739736-Registered on the 14th of November 2018- Retrospectively registered-

https://clinicaltrials.gov/ct2/results?cond=&term=NCT03739736&cntry=&state=&city=&dist

**Supplementary Information:**

The online version contains supplementary material available at 10.1186/s13033-022-00542-x.

## Introduction

Zambia has a long-standing history with tuberculosis (TB) and efforts to eradicate it date back to the 1960s [1]. Despite major progress in TB treatment and eradication, Zambia is still ranked among the thirty countries with the highest burden of TB globally [2]. This is due, in part, to the high prevalence of Human Immunodeficiency Virus (HIV) in the country. TB continues is one of the most significant co-infections for people living with HIV (PLWH) in Zambia, and accounts for a third of all deaths in this population [2]. Zambia has an estimated HIV prevalence of 11.3% amongst adults aged 15–49 and an approximate HIV/TB co-infection rate of 23.8% [3, 4].

Mental health conditions affecting people with TB include general psychological distress (which may itself be overwhelming and disabling) and conditions that can be diagnosed according to established classificatory systems (e.g. International Classification of Disease (ICD)), for example, depression and anxiety disorders. In general, mental health conditions that cause significant distress and functional and/or social impairment are regarded as mental illness (although individual, professional and cultural factors may affect the application of this label). Mental health conditions can act as a risk factor for TB, and amongst TB patients act as a barrier to TB management and eradication, by negatively impacting health behaviours such as diet, health care seeking, medication adherence, and/or treatment completion [5]. TB can in turn act as a risk factor for mental health conditions including psychological distress, depression and anxiety [6], as a result of morbidity and reduced quality of life [7, 8], duration and side effects of treatment [6, 9], social stigma attached to the illness [5, 8, 10, 11], fear of transmitting TB disease to others [6], and other potential comorbidities (especially HIV) that are associated with TB [12]. In this way, TB and mental ill-health share a syndemic relationship; each increases risk and exacerbates morbidity in the other. This creates a vicious cycle for those with co-morbid presentation of both, negatively impacting health and wellbeing [5].

As seen in other settings with high burden of TB, efforts to eradicate the disease in Zambia have not focused on the complex relationship between TB and mental health [13, 14]. The lack of attention given to mental health in TB care could be attributed to nascent mental health landscape in Zambia, which experiences several service provision gaps, including services being highly centralized, with few trained mental health personnel [15, 16].

Although the figure varies from one study to the other, the prevalence of mental health conditions was found to be high amongst TB patients. A 2020 global meta-analysis estimated a pooled prevalence of depression of 49.2% from 25 studies [17]. A significant proportion of the studies (8/25) in the meta-analysis were from sub-Saharan Africa, specifically three were from Nigeria with prevalence of depression ranging from 27.7 to 45.5%, one from Cameroon with a prevalence of 61%, and four from Ethiopia with a range of 31.1% to 54%. Similar to Zambia, the represented countries all have a high TB burden. The few studies looking at mental health and TB in the Zambian context also highlight potentially high levels of distress in TB patients. For example, a 2013 study conducted across 16 primary health centres in Zambia found 30.9% of 231 TB patients expressed suicidality, with 3.4% having attempted suicide at least once [18].

The 2015–2035 World Health Organization (WHO) End TB Strategy strongly advocates for the integration of mental health and TB treatment [19], however low- and middle-income countries (LMICs) often lack the trained mental health personnel needed to deliver such services [20]. Sweetland et al. [20] discuss how a task shifting model established within TB care in many LMICs, is well placed for the detection and treatment of mental health conditions, like depression, in TB patients. They emphasise that the bulk of TB care in LMICs, including TB screening and follow-up of patients, is conducted by community health workers, and training this cadre of health workers on how to detect and manage mental health conditions among TB patients would be an efficient way of integrating mental health service into the current TB infrastructure [20].

Health workers and other TB stakeholders, such as home-based care providers and public health institutions, are at the core of TB service provision, and their perspectives and understanding of mental health and TB, and the delivery of care, are integral to implementing an integrated model. Literature from the region highlights, for example, how negative attitudes of health workers serve as a barrier towards treatment for both TB and mental health conditions among patients [21–23]. To the best of our knowledge, there has been no work done to understand how TB stakeholders and health workers in Zambia understand the possible causes and implications of mental health conditions among TB patients, and how they currently navigate mental health screening and treatment in TB patients. We define TB stakeholders as individuals or organisations that work directly with TB control or management. This study sets out to explore: the conceptualization of mental health and TB amongst TB health workers and stakeholders; the causes of mental health conditions in TB patients as understood by health workers and stakeholders; and mental health screening and treatment practices delivered by TB health workers in Zambia.

## Methods

The study draws on qualitative data collected in 2018 as part of the Tuberculosis Reduction through Expanded Antiretroviral Treatment and Screening for active TB trial (TREATS). TREATS is a follow-on study from the Population Effects of Antiretroviral Therapy to Reduce HIV Transmission (PopART) Trial, a randomised control trial that aimed to investigate the impact of combined community level TB/HIV interventions on HIV incidence [24]. PopART was conducted in 21 communities—12 in Zambia and 9 in South Africa.

The aim of TREATS is to measure the impact of the PopART intervention on TB incidence and prevalence. TREATS is being carried out in the same 12 communities as PopART in Zambia. The TREATS qualitative inquiry, where this data was collected, was conducted in four intervention and four control communities. The qualitative work aimed to comparatively describe how TB patients experienced the PopART intervention and document popular understanding of TB and TB stigma in these communities. Data was collected by a social science team consisting of seven field-based research assistants. The research assistants were development studies [5] and social work [3] graduates [4 males and 3 females], who were guided by three experienced social scientists who provided training, de-briefing and field support (first, second and last authors). The social scientists are from different disciplines (economics, education, and anthropology) and were guided by the third author who is a psychiatrist with extensive experience in the region. Due to the sensitive nature of the data being collected, researcher assistants were trained to be sensitive to participants and reflective of their own biases around mental health conditions, how their identity impacts participants and the resulting implications on the data collected.

In each of the eight communities, we conducted Focus Group Discussions (FGDs) with local Neighbourhood Health Committees (NHCs) working in the field of TB. The NHCs are community-based groups formed under the guidance of health personnel that play a key role in health planning and budgeting activities. They serve as a link between the community and the health facility, advocating for disease prevention and control in their communities. We also conducted FGDs with TB stakeholders directly delivering TB services, including screening, testing and follow-up care for TB patients in our eight study communities. TB stakeholders included government organizations such as schools and clinics, and non-government organizations such as churches, public health institutions, and home-based care organizations.

FGDs were selected to explore the perspectives of various stakeholders in the field of TB. Locations most convenient for participants were organised prior to the discussions, and venues were chosen to maximise the privacy of participants. Open-ended questions were guided by a semi-structured interview guide to encourage wide-ranging perspectives. Each FGD was facilitated by a moderator who guided the discussion and an assistant moderator who assisted with facilitation and note taking. Each FGD was approximately 1.5 to 2 h long.

Data were also collected via in-depth interviews (IDIs) with TB health workers, exploring mental health conceptualisation and the experiences of mental health treatment and management of TB patients. The majority (8/9) of the health workers recruited were nurses and one was a health technician. All worked predominantly with diagnosed TB patients, providing medication and follow-up care in their respective clinics. Interviews lasted between 45 min to 1.5 h. Interviews were conducted in a private and comfortable location for the participants.

FGDs and IDIs interview guides included questions on mental health guided by the literature. Areas of inquiry included the understanding of mental health in the Zambian context, the drivers of mental health conditions during TB diagnosis and treatment, the implications of mental health conditions for TB patients’ quality of life and TB treatment outcomes, and availability and accessibility of mental health services for TB patients.

### Recruitment

The FGDs were conducted with a maximum of 10 to 12 participants. Maximum variation sampling was employed, with a spread based on age, sex, and occupation. Participants were chosen with the aim of encouraging varied viewpoints while keeping the atmosphere and power relations comfortable enough for everyone to contribute. Purposive sampling was used to recruit health workers for the IDIs. The eligibility criteria for health workers and the NHC participants included providing key TB services in the clinics from the eight TREATS communities during the time of the PopART trial through data collection for this study. NHC members were recruited through the help of community mobilisers, who serve as the link between the TREATS study and the community members. Clinic-based TB treatment supporters were also used to help identify members of the NHC. Subsequently, the TB stakeholders in each community were identified through FGDs with the NHC.

Table 1 provides a summary of data collection activities and description of participants.

**Table 1:** Table of participants

Activity	Participant type	Characteristic	
In-depth interviews	TB health worker (N = 9)	Male	2
		Female	7
		Age range	25–58
Focus group discussion	NHC (N = 96)	Number of activities	9
	Stakeholders (N = 57)	Number of activities	9

### Data analysis

IDIs and FGDs were recorded and transcribed verbatim. Data was analysed thematically using a coding framework developed from the TB and mental health literature. Further codes were iteratively added during the analysis. Data was double-coded by the first and second authors using ATLAS.ti software. Any discrepancies with themes emerging from the data were resolved with correspondence with the last author and securing common consent among the first, second and last authors.

## Results

The results are presented under three broad themes. The first explores TB stakeholders’ and health workers’ conceptualization of mental health and the impact of mental health conditions on TB patients experiences and overall wellbeing. The second explores causes of mental health conditions in TB patients, as understood by TB stakeholders and health workers. Thirdly, the results explore how TB health workers screen and treat mental health conditions in TB patients.

### Conceptualisation of mental health and implications of mental health conditions for TB patients by TB health workers and stakeholders

There were two overarching definitions of mental health conditions presented by health worker and other stakeholder participants; *“Ku funta”* and *“Ku ganiza maningi”*, loosely translated to “*madness*” and “*thinking too much*”. “*Madness”* was more equivalent to psychotic symptoms of mental illness while “*thinking too much*” was similar to symptoms of depression and anxiety. Participants also identified implications of mental health conditions on TB patients, namely suicide, poor adherence, and disruption to family dynamics.

#### Conceptualisation of mental health and mental health conditions

##### “Madness” (*“Ku funta”)*

When initially asked about mental health and mental health conditions, most participants were momentarily discontented and often broke out into nervous laughter before responding. The terms “mental health conditions and mental illness” did not illicit definitions from health workers and TB stakeholders that were equivalent to a biomedical definition of the terms. Rather, *“Madness” (Ku funta)* was often the initial definition of mental illness stated by stakeholders. In the local context the word more often implies a characterological challenge or personality flaw rather than an illness. Taken further, a “*mad*” person in this context can also be called “*Chi Silu”*, which loosely translates into “a foolish or useless person”. Such conceptualisations of mental illness significantly devalue those experiencing symptoms and when used in dialogue, these definitions have strong stigmatizing undertones towards people with mental health conditions. For example, when asked to define mental health, one NHC FGD (Z3) said it was *“madness of the mind”.*

The symptoms of “*madness”* provided by stakeholders included: patients behaving irrationally, being short tempered and aggressive, hoarding items such as stones and animal skins, refusing to take their medication, “*delusional behaviour*”, extreme sex drive (particularly in men) or no sex drive at all, and memory loss.

##### “Overthinking or thinking too much” (*“Ku ganiza maningi”)*

When asked specifically about depression and anxiety disorders in TB patients, stakeholders and health workers had different ways of expressing these conditions. Some of the definitions included TB patients being the term “*emotional*” (sometimes with emphasis, for example, “*over emotional”, “emotionally stressed”),* but most commonly they were described as *“Ku ganiza maningi” (“overthinking* or *thinking too much”)* by most of the participants. Participants described TB patients who were “*over thinking* or *thinking too much”* as being overly sad and incessantly worried, with a tendency of isolating themselves.

#### Implications of mental health conditions

##### Suicide

Participants acknowledged the negative implications that mental health conditions had on the lives of TB patients; pinpointing an amalgamation of personal and social losses, including loss of self-worth and standing within their communities, which in some cases reportedly resulted in suicide.

*“They (TB patients) start seeing that they are nothing in the community, that there is nothing that they can even do. Because people have looked down on them, that is what now becomes of the person. Others end up killing themselves because of such thinking.” (FGD, NHC, Z3)*

##### Poor adherence

Participants further detailed how mental health conditions such as depression, anxiety and alcohol addiction sometimes led to poor adherence in their patients, particularly for those with poor social support systems.

*“I: In your opinion, do emotional and mental health problems affect the ability of TB patients to adhere to treatment? R: Yes because they will end up thinking about things that are not even in existence. As a result, they will not even trust the same medication that they are taking and, in the end, they will fail to adhere to TB treatment and later on they will become defaulters.” (IDI, Health worker, Female, Z7)*

In some cases, recalled by participants, mental health conditions were present before their patient’s TB diagnosis and served as a barrier to engagement with TB treatment, which unfortunately resulted in death for some TB patients, as seen in the example below:

*“R: According to the information we got from his parents, he was already depressed ever since his wife left him after he got fired from his Job, so when he was even found with TB he refused to start treatment even when his parents escorted him here to the clinic. We educated him, explained to him, he still refused to start treatment. I: Ok what about the other person who died? R: The other person who died, we used to talk to him, but he didn’t accept that he had TB and he loved drinking alcohol too much. He would come and collect drugs on time but would just go and pack the medicine at home and would not drink it.” (FGD, Stakeholders, Z4)*

Despite acknowledging that TB patients sometimes defaulted because of a mental health condition, participants still felt a sense of responsibility for their patient’s challenges in adhering to their treatment. For example, there was a sense of frustration in a stakeholder who described feeling ignored and like a failure when their patients defaulted and died.

*“We have seen them (TB patients) dying because of poor adherence. You find that when you advise them not to drink, they are found drinking all sorts of beer. That is one thing that I have seen where we have failed on our side as helpers to these people in the community. It’s like they are ignoring us. Sometimes they come to a point where, I don’t know whether it’s stigma or self-denial, where they reach a point of let it (death) come, I die. We really feel our work is not being heard because the people that you have been informing for some time will die and when you look back, you ask yourself: ‘we have been working with that person, we have been going to that person, but how has that person defaulted?’” (FGD, Stakeholder Z8)*

##### Disruption of household dynamics

Participants noted that psychological distress and mental health conditions in TB patients also negatively affected relationships within the household. They described distressed TB patients, particularly men, as aggressive and short tempered and, in some cases, suspicious of their spouses. These feelings sometimes resulted in violence towards women and was destabilising for the household.

*“Like what they have said, men are the most difficult, because they feel life has ceased and it will not be the same again. So, they are short tempered such that most of the times women don’t stay well. That’s why you see women leaving, it’s because of the behavior the man begins to portray when they are sick. They refuse medicine, sometimes they become very upset, and they do not want to take medicine. So, when their wife tries to force them to take the medicine, they would even want to beat their wives, so this causes the women to want to run away from what is happening with their husbands.”* (FGD, Stakeholders, Z7)

Participants highlighted both personal and social implications of psychological distress and mental health conditions in TB patients, including death through suicide and poor TB treatment adherence. In some cases, stakeholders and health workers reflected on the death of TB patients as personal failings in their duty despite acknowledging the role that mental health conditions played in these deaths.

### Causes of psychological distress and mental health conditions in TB patients as understood by stakeholders and health workers

According to TB stakeholders and health workers, psychological distress and mental health conditions in TB patients was either caused by physiological factors or social and economic factors. Physiological drivers were more associated with “madness”, while social and economic factors were associated with depressive symptoms. Stated physiological factors included location of TB in the body and side effects of TB medication. Social and economic drivers included loss of income resulting from TB morbidity, TB stigma, and misinformation or lack of adequate information about TB transmission and treatment, compounded by a fear of death.

#### Physiological factors

##### Locations of TB in the body

There was a belief among some stakeholders that TB causes *“madness”* due to physiological factors, such as “*TB bacteria entering the brain”* of the patient.

*“It depends on the area that the TB germ goes to live in the body. If its TB of the brain, it can make the person mad. They would be looking mad.” (FGD, NHC, Z3)*

##### Anti-TB medication

A minority of participants attributed mental health conditions in TB patients to TB medication, either as a direct side effect of the medication or because of the other side effects resulting from TB medication.

“The side effects of the medication make them (TB patients) experience mental health challenges.” (FGD, NHC, Z10)

#### Social and economic factors

##### Loss of ability to earn a living

Participants discussed the significant economic implications of TB, as a source of psychological distress for many TB patients who mostly work in the informal sector meaning their ability to earn was dependant on their ability to work. Participants stressed that few of their patients had the liberty of paid sick leave. Below, a member of a NHC describes the negative impact of TB on their patients’ income and the ripple effect this has on a patient’s family and mental health:

*“He (TB patient) is the one who goes for work, right, then he is diagnosed with TB, and he is told that he can’t go for work. You see, these jobs that they (TB patients) do, unless they report for work, they will not get paid, if they don’t report for work they won’t be paid. So, this adult will be thinking a lot… ‘how I am going to feed my children’. If it means going to the fields to farm, he is not able to because of the disease, so he will have a lot of thoughts about how to look after the family while he is sick. So, they have double problems, planning for the family and looking after themselves while they have the disease…...that’s where I see agony to be biggest.” (FGD, NHC, Z12)*

##### Stigma and discrimination

Most stakeholders and health workers believed that TB stigma was a major driver of psychological distress and mental health condition in TB patients. One stakeholder described stigmatization as *“mental torture” (FGD, NHC, Z3)* that would eventually result in “*madness”* in TB patients*.* Another FGD participant went as far as to state that in the absence of stigma, TB patients would not develop depression.

“If they (TB patient) are not being stigmatized they can’t go through depression.” (FGD, NHC, Z4)

The stigma, that sometimes resulted in discrimination, that most participants alluded to was experienced most commonly at household level, from family members or close friends living with TB patients. Forced isolation was a commonly discussed form of discrimination; for example, participants described how some caregivers make TB patients use their own eating utensils and bedding or force them to sleep in separate rooms.

*“Another thing that contributes (to* psychological distress) *is how those (family members) with TB patients behave. They avoid sitting near them (TB patients) ... Even eating with TB patients, they refuse saying, ‘you might contract TB’.” (FGD, NHC, Z7)*

Another participant in the same FGD went on to share a personal experience of how such behaviours led to their brother contemplating suicide whilst receiving TB treatment.

*“I have a younger sibling, his name is X, he had TB. When he had TB I used to stay with him and my in-laws… my aunt used to treat my brother in a way similar to what we discussed, where she would give him his own cup, plate and his own room... he used to have worries because of this treatment. He reached an extent of saying I would rather hang myself instead of me staying alone, there is no one close to me, no one even chats with me.”(FGD, NHC, Z7)*

As well as stigma from household members, participants told us that TB patients experience stigma in health care settings, particularly from health workers providing non-TB services. The negative attitudes of health workers towards TB patients were attributed to the fear of contracting TB and inadequate training about TB in general. Stigmatizing behaviours towards TB patients by health workers included talking to TB patients rudely and denying them health services.

*“R: The pharmacist said, ‘no no no, he (TB patient) is infectious, he will infect others so you should not be allowing him to come here, he should just stay at the TB corner. The peers should come and collect his drugs on his behalf…’. That is what happened.*

**I:** So, do health workers stigmatise TB patients?

**R**: They stigmatised that one.

**I**: *Nervous Laugh* So they do.

**I2**: Why do you think other health workers would stigmatise them?

**R**: They are scared of contracting TB.” (IDI, Health Worker, Z7)

##### Misinformation about TB

Limited understanding of TB transmission was discussed as another common driver of psychological distress and mental health conditions amongst TB patients.

*“I think it (*psychological distress) *all goes back to lack of knowledge and understanding of the TB infection. To some people, once they are diagnosed with TB, they will feel emotionally defeated and challenged. They will feel that what they have is something that will never be cured.” (IDI, Health Worker, Female, Z2)*

Participants also highlighted how some TB patients believe that TB is incurable and how this misinformation often contributed to psychological distress and mental health conditions.

*“The reason why they (TB patients) think too much when they have TB, is that they think that they won’t get well and they will die.” (FGD, TB Stakeholder, Z3)*

This lack of understanding about TB treatment was underlined by the misunderstanding of many TB patients as to the cause of the disease, informed by popular assumptions and beliefs. According to participants, patients mentioned that TB was contracted through excessive drinking or smoking, “promiscuous” behaviour (having sex with multiple partners) or sexual contact with a woman who had had an abortion. These popular assumptions and beliefs served as drivers of TB stigma, increasing feelings of shame and reducing perceptions of self-worth amongst TB patients. Participants stressed how these beliefs made it harder for TB patients to understand how they were infected with TB, illustrated by the stakeholder’s experience below:

*“After seeing her TB results in red pen stating smear positive and being told that she was found with TB and had to start drugs, she started crying saying, ‘I don’t sleep with men, am not a prostitute, how can I be found with TB…’” (FGD, TB Stakeholder, Z8)*

Additionally, participants noted that TB patients often associated TB with HIV, with many of their TB patients automatically assuming that their TB diagnosis meant that they were also HIV positive. This association between the two conditions was often emotionally distressing for TB patients.

*“Others had beliefs to say when you have TB, and definitely when you start TB drugs, then you will have HIV; those were the misconceptions that they had…TB patients think that when they start taking TB drugs people will know that they are HIV positive.” (FGD, TB Stakeholder, Z8)*

Participants did note, however, that a genuine experience of TB and HIV co-morbidity did present an extra emotional burden on TB patients. TB health workers bore witness to how emotionally challenging it was for patients to accept and cope with having both TB and HIV. For some of their patients, the co-morbidity, underscored feelings of denial and depression and could lead to death.

*“They (two TB patients) were depressed because they were told that they had TB and HIV. The one who was from area X was very much in denial. He refused to believe he could have TB or HIV because he claimed he never had sex with women. That is how he died. Then the other one had a wife who was pregnant. He also refused to accept that he had TB. A lot of them believe when you have TB then you automatically have HIV. He refused to accept it. We tried by all means to help and counsel him. When you are around, he would accept to come (to the clinic) but when you leave his home, he would change his mind. That is how he died.” (FGD, NHC, Z2)*

TB health workers and stakeholders provided a detailed understanding of the causes of psychological distress and mental health conditions among TB patients during TB investigation and treatment. Participants placed more emphasis on the interactions between social and economic level drivers as compared physiological causes of psychological distress and mental health conditions.

### Identification and treatment of mental health conditions in TB patients

Participants told us that they did not receive any in-depth training with regards to mental health and they do not have a standard method for identifying mental health conditions in TB patients during routine TB care. Treatment options in cases where participants suspected a mental health condition in their TB patients included adherence support, encouragement and referral to the main psychiatric hospital.

#### Screening and training

##### Mental health screening

TB health workers and stakeholders told us that mental health screening is not part of routine TB treatment in Zambia. Both diagnosis and treatment of mental health conditions were reliant on the health workers’ interpretation of patients’ emotional state. For example, some health workers said that they would raise mental health issues with specific patients if the patient looked visibly upset by the news of their TB diagnosis or appeared to be isolating themselves from others.

“**I:** Do you regularly provide emotional support to TB patients in your work?”

*R: Not so often, but once there is a new client, we give ample time to that client to express themselves and ask questions where they are not sure. We then explain to them according to the questions they are asking. We give them time to talk and ask questions.” (IDI, Health Worker, Female, Z8)*

##### Training

Most health workers interviewed stated that they had not received any in-depth training regarding mental health and illness. Some had undergone short psychosocial counselling courses, but such courses were not targeted towards supporting chronically ill patients with depression and anxiety. For those that had received mental health training, they had rarely received regular refresher training or support.

*“Eh… I wanted to say that we do not have health workers who are professionally trained in dealing with depression. They are trained in general counselling, where they talk on the surface and try to calm the person (distressed patient). We don’t have someone who’s trained specifically for depression or organizations that have come in to help on matters of depression.” (FGD, TB stakeholder, Z3)*

#### Treatment of mental health conditions in TB patients

##### TB knowledge and adherence support

Treatment offered by health workers for psychological distress and mental health conditions is often focused on a discussion around TB treatment, with the belief that distressed patients just need encouragement and knowledge about their condition. Most of these discussions are provided by the nurses in charge of TB treatment and by TB treatment supporters (community volunteers). TB treatment supporters also provide education on TB, informing patients and the community about TB causes, transmission, and how to take care of themselves when diagnosed. This health education places emphasis on the importance of adherence to medication to cure TB and often try to dispel misconceptions about TB.

*“Every time they (TB patients) take their drugs, we make them sit here and then we ask, ‘what is your problem?’ Then we try to explain what TB is and give them information about TB. We ask the patient, ‘do you really understand this disease you have?’, ‘where you contracted it?’, ‘how do you feel?’, ‘where do you feel you got it?’, and they will try to explain to us and then we chip in and give the information if there are any myths and misconceptions.” (IDI, Health Worker, Female, Z8)*

##### Referral to psychiatric hospital

Some health workers acknowledged that they were not equipped to deal with mental health conditions. For example, when asked about psychological distress or mental health conditions in her patients, one nurse (IDI, Health Worker, Z7) simply stated, “*Hmm, I am not a mental nurse… I don’t have a psychiatric paper (degree).”* As a result, some health workers and stakeholders opted to refer emotionally distressed patients to the country’s main psychiatric hospital.

*“…..at times we refer those people (TB patients), in case it becomes complicated, like when they start becoming mentally ill….” (FGD, TB stakeholders, Z7)*

TB health workers acknowledged that they were not well equipped to handle the mental health needs of TB patients dealing with psychological distress or mental health conditions.

## Discussion

This research has explored conceptualisation, implications, and drivers of psychological distress and mental health conditions among TB patients in Zambia as understood by TB stakeholders and health workers. Insights into current mental health screening and treatment practices have also been highlighted by participants. It is clear from our findings that TB stakeholders and health workers are mindful of the drivers and implications of psychological distress and mental health conditions among TB patients. There are limitations in participants conceptualisation of mental health and mental health conditions, which in part are due to inadequate training and guidance on mental-ill health management in comorbid TB patients. Integrating mental health training and support into TB clinics may offer a solution.

The TB stakeholders and health workers we spoke to in our study conceptualised mental health conditions into two distinct categories: madness and overthinking. “*Madness” (Ku funta)* was often the initial way in which TB stakeholders and health workers described potential mental health conditions in TB patients and was more equivalent to psychotic symptoms caused by physiological factors, such as TB entering the brain or TB medication. Although there is some literature that suggests TB infection [5] and TB medication can lead to neuropsychiatric complications in TB patients, these complications are rare; a global meta-analysis calculated a pooled estimate of 1.1% (95% CI 0.2–2.1) for central nervous system related adverse drug reactions in TB patients [25]. Our findings reveal that this conceptualisation of mental health conditions was inundated with stigmatizing undertones affirming the work of other studies exploring the attitudes towards mental health conditions in Zambia [16, 21, 26, 27].

Our researchers were not impervious to these attitudes. Despite comprehensive training in mental health conditions and stigma prior to data collection, research assistants still used stigmatizing language such as *“(ku funta) madness, and sickness of the mind”* in reference to mental health conditions, when conducting interviews. This highlights how deep-rooted mental health conditions biases are in this context and the potential challenges that may arise in attitudinal shifts towards mental health conditions. More research around the understanding of mental health and illness in this context would provide guidance of how to talk about mental health conditions in a sensitive and context appropriate manner. Stigmatizing attitudes towards mental health conditions are not unique to Zambia and are prevalent throughout the region [28–30]. They are, in part, driven by traditional beliefs in supernatural causes of mental health conditions, such as witchcraft [26, 30]. In our findings however, TB occupied a parallel space to witchcraft, providing another explanation as to the cause of “*madness*” in TB patients.

There are profound negative implications of these stigmatizing attitudes for TB patients dealing with mental health conditions on both their quality of life and health seeking behaviours [6]. TB alone is a highly stigmatized condition in Zambia [31] and TB is strongly associated with HIV, again heavily stigmatised in this context [32]. Thus, TB patients dealing with mental health conditions could experience a convergence of multiple stigmas. It is vital that the government and service providers tackle the multiple stigmas associated with mental health conditions, TB and other stigmatized conditions. The health-related stigma framework, developed by Stangl et al. [33] offers guidance in tackling stigma brought about by mental health conditions and TB. It acknowledges and addresses parallels in drivers and consequences of stigma across different conditions and recommends a cross-cutting approach to addressing stigma. Research focused on this framework and the creation of context sensitive interventions addressing intersectional stigma would be highly beneficial in this context.

The attitudes towards mental health conditions in Zambia are present not only among community members, but also at policy level, as evidenced by the 1951–2019 Mental Disorders Act use of archaic terms such as *“idiots, imbeciles,* and *invalids”* in reference to patients with mental health conditions [15]. Stigma towards mental health conditions could be partly addressed by the new Mental Health Act passed in April 2019. Among other things, the Act aims to increase awareness of mental health and tackle mental health stigma [34]. Implementation of this act will require government commitment, funding and coordination of community awareness activities and comprehensive changes throughout public administration, if understanding is to be improved and stigmatising attitudes reduced.

Aside from “*madness*”, TB health workers and stakeholders also described TB patients in psychological distress as “*overthinking* or *thinking too much”,* a description similar to other countries in the region [35–38]. Our participants’ conceptualization of “*overthinking* or *thinking too much”* has overlaps with depression; for example, both are characterised by sadness/low mood, isolation, and suicidal ideation. However, there are significant differences between the two, namely, *“overthinking* or *thinking too much”,* unlike depression, was not considered to be an actual illness by participants and was thought to be alleviated through provision of adequate information about TB and rendering encouragement to TB patients who appear distressed. The conceptualisation of “*overthinking* or *thinking too much”* as a social problem, which consequently requires a social solution, is echoed in work from the region. For example, in work with HIV positive women in South Africa, HIV infection was not considered to be the cause of their psychological distress but rather social factors such as stigma, poverty, and stressful life events resulting from their condition were to blame [39]. Accordingly, social support emerged from participants as the most appropriate intervention for psychological distress or mental health conditions [39]. Psychosocial interventions offer non-pharmacological solutions and are instead focused on psychological or social factors of psychological distress and mental health conditions. They are well-evidenced to be effective for people with mental health conditions and co-morbid physical conditions, especially in low-resource settings [40]. Mental health integration into TB care would benefit from research exploring psychosocial interventions among TB patients in this context, given our findings of limited support options for people with mental health conditions in Zambia and limited training of health care staff.

As well as limited mental health support for people with mental health conditions in Zambia, our findings also indicate a dearth of standard screening and diagnosing mechanisms for TB patients suspected to be dealing with mental health conditions. Both diagnosis and the consequent treatment options were reliant on health workers’ perceptions of the patient. When thinking on the need for better diagnosis and intervention, it is important to recognise that Zambia is extremely under resourced in relation to trained mental health personnel [15]. As Sweetland et al. [20] argue, a context such as Zambia could lend itself well to the WHO Mental Health Gap Action Programme (mhGAP) model. mhGAP utilises a task-shifting model, training non-mental health specialists in detection and management of ten priority disorders including psychosis, alcohol and drug use, depression, and suicide [41]. This model places emphasis on pharmacological treatments, while providing some brief structured psychotherapies that can be delivered by non-specialists [41]. In Zambia, such training could be provided to both clinic-based health workers and community health workers, who are at the helm of TB management. Community health workers provide services such as community TB screening, contact tracing, and follow-up and monitoring of TB patients in the community. Their established position within local communities and the primary care structure would allow for a more contextually sensitive understanding and delivery of mental health services and can alleviate the need for specialist mental health personnel.

This model of training non-specialised health workers in detection and treatment of mental health conditions has proved to be somewhat effective in the region. Evidence on task-shifting models has demonstrated improved knowledge about mental health conditions and improve confidence in identifying mental health conditions among clinic based [42–44] and community based [45] health workers, as well as significant reductions in symptoms of mental health conditions among patients in primary care [46]. Evidence has shown the benefits of similar interventions for non-TB groups in Zambia, and it may prove appropriate for TB patients [47]. There are possible concerns with this approach, including a potential over use of pharmacological solutions to address psychosocial challenges, resource constraints affecting adequate supervision of non-specialised mental health staff [48] and the need to appropriately motivate and compensate community health workers without relegating their dedication and constantly evolving roles and responsibilities to bouts of altruism [49]. That said, evidence suggests such a model may be appropriate for mental health in TB patients in a Zambian context, especially when integrated into the TB care system, where health workers are given very little support and training on mental health, despite the number of people they meet needing support. Future research should focus adoption and evaluation of mhGAP or similar task-shifting models, following well-evidenced adaptation processes, feasibility studies and pilot evaluations, to inform a culturally and contextually appropriate intervention [50]. Any intervention developed for TB patients should incorporate improved information on TB cause and transmission, having seen our participants describe widespread assumptions that have an impact on psychological distress and self-stigma. Developing an intervention that addresses both the underlying causes of mental health conditions in TB patients, as well as alleviation of prevailing symptoms, will be needed to reduce the burden among this population in Zambia.

The findings of this study come at a timely juncture, as the world of TB contemplates ways of integrating mental health screening and treatment into the TB care cascade. The work provides insight into how health workers and stakeholders working in the field of TB understand the intersection between mental health conditions and TB in the Zambian setting, highlighting baseline knowledge and gaps. Recommendations for further research based on our findings include: contextual understanding of mental health conditions; stigma interventions tacking intersectional mental health, TB and HIV stigma; training for health facility and community-based health workers on mental health screening and treatment; and an exploration and evaluation of psychosocial interventions with TB patients screened for mental health conditions in the Zambian context. Furthermore, in context of these findings, it is worth addressing the social underlying causes of mental health conditions in TB patients including improving social security nets for economically vulnerable TB patients.

### Limitations

Despite training, some research assistants in the study maintained stigmatising attitudes towards people with mental health conditions, which were evident in their interpretation/communication of the mental health questions to participants. The major strengths of the study were the large sample size and geographical reach of participants which spanned across eight urban communities in Zambia. This allowed for exploration of diverse experiences and views around mental health and TB from a sample with national level representation.

## Conclusion

TB stakeholders and health workers are cognisant of the increased risk of psychological distress and mental health conditions among TB patients, although their understanding of mental health conditions often comes through stigmatizing. Health workers generally lack the toolkit to adequately detect and treat mental health conditions in their patients and could benefit from mental health training. The mental health training and sensitization for health workers should aim to increase knowledge about mental health and chronic illness, while shifting negative attitudes around mental health conditions. Task-shifting models may represent an appropriate method to integrate a standardised pathway of care for TB patients with mental health conditions in TB clinics. Interventions must include contextually appropriate identification and treatment methods, and efforts must be made to address the social drivers of psychological distress and mental health conditions, including TB stigma and economic vulnerability.

## Supplementary Information

**Additional file 1.** Stakeholder analysis interview guide.

**Additional file 2.** TB health worker in-depth interview guide.

## Data Availability

The data used or analysed during the current study are available from the corresponding author on reasonable request.

## Abbreviations

FGDs: Focus group discussions
ICD: International classification of disease
IDIs: In-depth interviews
HIV: Human immunodeficiency virus
LMIC: Low- and middle-income countries
LSHTM: London School of Hygiene and Tropical Medicine
MhGAP: Mental health gap action programme
NHCs: Neighbourhood health committees
PLWH: People living with HIV
PopART: Population effects of antiretroviral therapy to reduce HIV transmission
TB: Tuberculosis
TREATS: Tuberculosis reduction through expanded antiretroviral treatment and screening for active TB trial
WHO: World Health Organization
