# Supplementary material for: Conceptualization, detection, and management of psychological distress and mental health conditions among people with tuberculosis in Zambia: a qualitative study with stakeholders’ and TB health workers
Source: Int J Ment Health Syst. 2022 Jul 12;16:34. doi: 10.1186/s13033-022-00542-x (PMC9275023; doi:10.1186/s13033-022-00542-x)
Supplement: Supplementary file 1 — Additional file 1. Stakeholder analysis interview guide. [file 13033_2022_542_MOESM1_ESM.pdf]

Tuberculosis (TB) reduction through expanding antiretroviral therapy for Tuberculosis screening (TREATS)

Stakeholder Analysis Discussion Guide/ IDI

Purpose: To describe the impact of PopART on the provision of health services for people living with TB. (Please note that this guide can also be used as interview guide for stakeholders who missed the discussion)

Objectives:

- To describe popular understandings of TB among stakeholders in PopART communities
- To describe the strategies and processes supporting implementation of TB-related services in the communities
- To explore perceptions TB treatment and care from stakeholders' perspectives
- To describe the influence of TB-stigma on the uptake and adherence to TB treatment from a stakeholders 'perspective
- To explore the interaction between the stakeholders and the potential interactions between stakeholder and the TREATS study.

Form of data recording: (1) Audio-recording of all talk from "Preamble" to "Closing". (2) Notes of key points per topic area handwritten by the facilitator into a printed copy of this document. (3) Photographs of each of the activities. (4) Handwritten notes by participants during the course of the discussion.

Expected time needed: 45 minutes

Date activity conducted: ____________________________

Place conducted: ___________________________________

Time period: ______________________________________

Preamble (to be read by facilitator): Today is the (insert date [day xxth Xx xxxx]) and it is (insert time XX:XX). This is a discussion with an implementing staff at (XXXX) Clinic. Thank you for your time. All information collected here will be reported anonymously. May I remind you that we are audio recording this discussion and ask that you speak loudly and clearly. As the facilitator, I will also be taking some notes. Do you have any questions before we begin?

INTRODUCTION [2 minutes]

We have selected all of you to represent your community because of the role you play related to provision of TB services. We ask you to be free to share as much as can during the discussion/ meeting.

Topic Area 1 **– Description of Stakeholder**

(Facilitator to read bolded text below and elaborate with prompts at their discretion)

Activity: Stakeholder Profile

Open the meeting with a short 'presentation' on what TREATS is and allow for time to respond to questions the stakeholders may have.

**Variable**	**Task**	**Objective**
Name	– Distribute plain coloured cards (half of A4 size) to each stakeholder participant.<br>– Ask them to write the name of the organization they are representing on top of the paper.<br>– Below it ask them to write the year they started working in the PopART community.	Identify stakeholders
Period	– Collect the papers from the participants and stick them on a wall according to the year they started working in the community.	Period service delivery
Involvement	– Give them A4 size paper to write/list the activities or services they provider in bullet form.<br>– Collect the papers and stick them below the earlier paper with name of stakeholder and the year –leave small space in-between.	Whether TB service delivery is primary objective
Interest	– Ask them to list TB activities or services they provide in the community, or what they do to assist their clients/patients' concern on TB (List TB activities/services).<br>– After they list down, collect the papers and place them under the organisations'' names on the wall.	Whether TB service delivery is secondary/tertiary objective

Influence	– Ask them if they work together with other organizations in any way on TB related activities (should write this on separate A4 papers).<br>– Ask them to write about how they think TREATS will influence their work; positive/negative?<br>– Ask them if they at all worked or collaborated with the CHiPs on TB activities.<br>– If any of the stakeholders did, ask them to list the role they played with the CHiPs (should write this on separate paper).<br>– List other stakeholders not present.	Influence of stakeholder on TB service delivery in the community.
Alignment	– Ask them to explain the groups, age, gender, and areas they work with/ from in the community.	Groups targeted and not targeted

After the stakeholder profile activity has been completed you may ask questions from the topic areas below to complement information gathered above. If it is not possible to gather all the required information for all the stakeholders, schedule specific meetings with the affected stakeholders as well as those that were not present.

Topic Area 2 – **TB specific activities**

1. **Do you do any TB related activities?** What do you do? Why? When do you do them? Do you work with the clinic? How long have you worked with the clinic?
2. **How has working in this community been**? Do you specifically work with TB patients? How do you identify them? Are there any specific places where these people are found? Do you find it challenging working with them? Why?
3. **Do you give any kind of support to TB patients**? What do you give? When do you give?
4. **Which TB programs have you done in the past**? What were you trying to achieve? What worked well/ did not work well? What do you think would have been done better? Which TB programme are you implementing now? Do you collaborate with other organisations in your implementation? Which organisations are those? Why?
5. **Are there any other organisations which conduct TB related activities you know of in this community**? Do they come from outside the community? Where do they work from? Who do they work with? What kind of activities do they do? How long have they worked in this community?

Topic area 4 – **Knowledge, Training and Experience**

1. **How have PopART activities (i.e. anything to do with PopART) affected activities (i.e. anything to do with TB treatment and care) in the community?** Do you think that PopART TB screening increased the number of people diagnosed and treated for TB at the clinic? Were any other people/organisations involved in the implementation of TB treatment provision? Please describe these people/organisations and what they did and how they contributed to TB services in the clinic and community.

2. **What has your experiences of delivering TB services in this community been like?** Are there any particular challenges to providing TB services in this community?
3. **Please tell me about any efforts to promote facility-based TB screening.** What are some of the initiatives that were implemented to promote TB treatment uptake? Which facility based health workers were involved and how? Over what period were these efforts implemented? How did you prioritise who to screen for TB? (prompt: Specific services access? Ages? Gender?) What do people say when they don't want to screen for TB?
4. **Were there any efforts that focused on particular groups of people?** Did you notice any differences in the age groups, or other characteristics, of those coming to the clinic? Did you get the impression that clients were more/less willing to screen for TB at certain times of the day, month or year?

Topic area 4 – **TB and Stigma**

1. **Has the way that people talk about TB in the community/ organization changed over the years?** What did they used to say about TB? What do they say now? Are people living with TB more or less stigmatised now than they were in the past? What do you think has contributed to the change?
2. **From what you know/ have seen, does the fear of being stigmatised stop people living with TB from seeking treatment/ care?** Can this also delay diagnosis of TB? If TB was not stigmatized, would people get treatment sooner? Why? Do you know of people living with TB who are afraid to ask their friends and family for help? Can you give an example of this?

We have now finished our discussion. Now, are there any questions you would like to ask us? Thank you very much for participating. We really value your time and the information and experiences you have shared with us.
