# Supplementary material for: Conceptualization, detection, and management of psychological distress and mental health conditions among people with tuberculosis in Zambia: a qualitative study with stakeholders’ and TB health workers
Source: Int J Ment Health Syst. 2022 Jul 12;16:34. doi: 10.1186/s13033-022-00542-x (PMC9275023; doi:10.1186/s13033-022-00542-x)
Supplement: Supplementary file 2 — Additional file 2. TB health worker in-depth interview guide. [file 13033_2022_542_MOESM2_ESM.pdf]

TUBERCULOSIS (TB)-Tuberculosis Reduction through Expanded Anti-retroviral and Screening for Active TB (TREATS)

TB Health Worker In-depth Interview Guide

<u>Purpose:</u>*To describe the impact of PopART on the provision of health services for people living with TB.*

Objectives:

- *To describe popular understandings of TB among health workers at PopART facilities*
- *To describe the strategies and processes supporting implementation of TB-related services at health facilities*
- *To explore perceptions TB treatment and care from a health workers' perspectives*
- *To describe the influence of TB-stigma on the uptake and adherence to TB treatment from a health workers' perspective*
- *To explore perceptions and experiences on the role of the CHiPs/ PopART in TB service provision*

<u>Materials needed:</u> *Map of community, map of clinic, coloured card cut into circles/ovals, marker pen, large white paper, informed consent forms, note book, pen, pencil, audio-recorder*

<u>Form of data recording:</u> *(1) Audio-recording of all talk from "Preamble" to "Closing". (2) Notes of key points per topic area handwritten by the facilitator into a printed copy of this document. (3) Photographs of each of the activities. (4) Handwritten notes by participants during the course of the discussion.*

<u>Expected time needed:</u> *Not more than an hour.*

Date activity conducted: ___________________________

Place conducted: _________________________________

Time period: _____________________________________

Administer Informed Consent – once/if completed continue.

Preamble (to be read by facilitator): Today is the (insert date [day xx[th] Xx xxxx]) and it is (insert time XX:XX). This is a discussion with a health staff working with TB at (XXXX) Clinic as part of the social science work of TREATS. Thank you for your time. All information collected here will be reported anonymously. If permissible, I would like to audio-record this discussion and I will also take some notes. We will also look at some maps together and discuss them. Do you have any questions before we begin?

(Facilitator to read bolded text below and elaborate with prompts at their discretion)

Topic Area 1 – Clinic and Community

1. **What is your position in the clinic?**

- What are some of your roles?
- How long have you been working with TB?
- How do you feel about working specifically with TB?
- Who else do you work with at this clinic when supporting TB patients?
- Do TB staff rotate through other departments?
- Have you ever done any specific training for TB? Please explain what kind of training.
- Please describe what you did yesterday at this TB clinic/corner?
    - *Probe: Starting from when you arrived at work to when you left, can you share with me what happens?*

2. **How long have you worked at this clinic?**

- What has changed since you started working here?
- What have been some of the biggest challenges while working here?

3. **What community/ies does this clinic serve?**

- What are some of the biggest challenges in this community?
- What health problems are found in this community, which are not a problem in other communities? How do they affect the clinic?

4. **Do you live in this community?**

- If no, where are you from?
- If yes, when did you move to this community? Why did you move to this community?
- Do any of your family members also live in this community?
- What is it like to work in the same community as where you live?
- Looking at this map of the community [*use map of community],* are there any places in the community that you consider more at risk of TB, like a 'TB hot-spot'? Probe for: particular housing/houses, particular areas, particular ethnicities in community, transport depots, bars/taverns/shebeens, video/gaming places, clinics, markets, schools. What makes a

place risky for TB? Probe for cooking stoves, alcohol habits, smoking habits, ventilation, crowding/congestion, darkness, dampness, sexual behaviour?

Topic Area 2 – Tell us about you

5. **Can you tell us more about your family?**

   - Household composition: Who lives with you? Are you married? Cohabitating? Single? Widowed? Divorced? Do you have any children? How old?
   - Household hierarchy: Who makes most of the decisions for the family? Who takes care of the home?

6. **Do you have any family members who have had TB before?**

   - When was that? Would you mind telling me a bit about this experience (who? Age? Gender? TB treatment? Outcome?)
   - Are you concerned about anyone in your family developing TB in the future? Why?

Topic area 3 – Knowledge, Training and Experience

7. **For ARM A sites only:** How have PopART activities (i.e. anything to do with PopART) affected TB activities (i.e. anything to do with TB treatment and care) in the clinic?

   - Do you know about the CHiPs? (Explain if not clear). What did they do in the households with TB when they went door to door? Do you personally know anyone diagnosed with TB through CHiPs?
   - Do you think that PopART TB screening in the households increased the number of people diagnosed and treated for TB at the clinic?
   - Since CHiPs stopped going door to door, has there been any change in the number of TB patients being diagnosed?

8. **For ALL sites: Are any other people/organisations involved in the implementation of TB treatment provision?**

   - Using this coloured card, [*have coloured card cut into circles/ovals and a large piece of plain white paper and markers. The paper should have a large circle on it representing the community and a clinic drawn in the middle],* could you name all these people/organisations on each card and place them on the circle representing the community and this clinic, and explain their relationship with this clinic, what they do and how they contribute to TB services in the clinic and community?
   - *For ARM C sites only:* In some other communities where we did PopART (explain if necessary what PopART is), we had CHWs going door to door doing HIV testing and counselling, and screening for TB. Have you ever had this type of initiative in this community? Please explain if you have. Do you think it is a good idea?

9. **Looking at the map of this clinic, can we discuss which departments you interact with when based at the TB corner/clinic?**

- Do you interact with: OPD, MCH, HIV, laboratory services, the pharmacy, the mortuary, the wards (Zambia only), any other areas?
- At the laboratory, how are they testing for TB? Probe: sputum, microscope, Gene expert, culture.
- In relation to TB drugs, what regimens do you give? What if someone has relapse TB? What if someone has multi-drug resistant TB?
- Please explain in some detail how you liaise with HIV services? Do all TB patients test for HIV? Do you test them here or refer them?
- Overall, what has your experience of providing TB services at this health facility been like?
- Have you experienced any challenges in delivering TB services? **Probe: Delay in results?** Shortage of medication? Shortage of staff? Long lines? Waiting periods at clinic? Lack of privacy? Congestion? Patients defaulting? Being asked for a transfer letter?
- Are there any things you would like to change about the way TB services are provided here?
- Are there any support systems for TB patients? Are there any food items given to TB patients?

10. What has your experiences of delivering TB services in this community been like?

- Are there any particular challenges to providing TB services in this community?

11. Are there any efforts to promote facility-based TB screening amongst staff and patients?

12. Were there any efforts that focused on particular groups of people?

- How did you prioritise who to screen for TB? (prompt: Specific services access? Ages? Gender?)

Topic area 4 – TB and Stigma

13. Looking at this map of the clinic*[show map of clinic]*:

- Where do TB patients feel comfortable?
- Where do TB patients feel uncomfortable?

14. Has the way that people talk about TB in the community/clinic changed over the years?

- What did they used to say about TB? What do they say now?
- Are TB patients more or less stigmatised now than they were in the past? What do you think has contributed to the change?

15. From what you know/ have seen, does the fear of being stigmatised stop people with TB symptoms from seeking treatment/ care?

- Can this also delay diagnosis of TB?
- If TB was not stigmatized, would people get treatment sooner? Why?

- Does stigma affect adherence to TB drugs?
- Does stigma ever cause death in your opinion?
- Do you know of TB patients who are afraid to ask their friends and family for help? Can you give an example of this?
- Do you think that people feel nervous about TB patients?
- Do you think that people feel pity for TB patients?
- Do you think that developing TB is a person's own fault?
- Do you feel angry towards TB patients ever?
- Do you think TB patients should ever be isolated?
- Do you think TB patients should be forced to take treatment if necessary?

16. **From what you know, do health workers stigmatise TB patients**?
    - Do some health workers stigmatise people living with TB?
    - How does it make you feel when TB patients cough while at the clinic?
    - Are you concerned about developing TB?
    - How do you manage that?
17. **If a health worker is also living with HIV, are they advised to take IPT to protect them against TB?**
18. **In your experience, are health workers that provide TB services stigmatized?**
    - Has this ever happened to you or someone you know? Could you tell us about it?
19. **How do you think ART has affected TB stigma? Has it made it reduce or increase?**
    - In your experience what is it like for TB patients who also have HIV?
    - Do you think it is hard to take two different sets of pills?
    - Do you think they face stigma for having both diseases?
20. **What could be done about stigma towards TB patients?**

Thank you so much for all your time. We really value you sharing your experience and opinions. Are there any questions you would now like to ask me
